# Fistula of the mitral-aortic intervalvular fibrosa in a patient with bacterial endocarditis: a case report and systematic literature review

**DOI:** 10.1186/s13019-024-02736-5

**Published:** 2024-05-28

**Authors:** Rafael Figueroa-Casanova, Juan D. Saavedra-Henao, Juan S. Figueroa-Laverde, Diego A. Beltrán-Gonzales, José G. Labrador-Rosales, Sara Eslait-Olaciregui, Carlos J. Pérez Rivera

**Affiliations:** 1 Cardiovascular Surgery Department, Clínica Avidanti, Tolima, Ibagué, Colombia; 2 Clínica Avidanti, Tolima, Ibagué, Colombia; 3 Universidad del Bosque, Bogotá, DC Colombia; 4 Universidad del Rosario, Bogotá, Colombia; 5 Surgery, Universidad El Bosque, Cundinamarca, Bogotá, Colombia

**Keywords:** Endocarditis, Mitral-aortic intervalvular fibrosa, Transesophageal echocardiography

## Abstract

**Background:**

A fistulous tract in the mitro-aortic intervalvular fibrosa (MAIVF) is a rare entity, which presents as a complication of endocarditis or surgical trauma. Generally, it is associated to a pseudoaneurysm of the MAIVF (p-MAIVF) or aortic abscesses. MAIVF fistulas could potentially lead to devastating complications and a high mortality rate. This condition is managed surgically, either by a percutaneous closure or an open surgical approach. Herein we report the complex case of a patient with a MAIVF fistula secondary to bacterial endocarditis. Further clinical deterioration was caused by severe aortic valve insufficiency and hemodynamic compromise, requiring surgical intervention.

**Case presentation:**

A 74-year-old male patient was admitted to a primary care center with complaints of malaise, asthenia, adynamia, hyporexia, and lower limb edema over the past eight days. His past medical history is positive for arterial hypertension and being monorenal. A transesophageal echocardiogram (TEE) was performed, exhibiting a 56% left ventricle ejection fraction (LVEF) and complicated aortic valve endocarditis. Surgical management through an open approach included vegetation resection, valve replacement, and closure of the MAIVF fistula. After completing antibiotic therapy, the patient was discharged without complications. During postoperative follow-up, the patient remained asymptomatic, and the control echocardiogram showed no signs of MAIVF fistula.4.

**Conclusions:**

The clinical case of a patient with a MAIVF fistula secondary to endocarditis by *Streptococcus Anginous* was presented. The fistulous tract was not associated to p-MAIVF or aortic abscess, findings which further deteriorate the patient’s condition and increase the likelihood of fatality. This case reinforces the importance of a prompt diagnosis through cardiac imaging and timely surgical closure of the defect.

**Supplementary Information:**

The online version contains supplementary material available at 10.1186/s13019-024-02736-5.

## Background

A fistulous tract in the mitro-aortic intervalvular fibrosa (MAIVF) is a rare entity, which presents as a complication of endocarditis or surgical trauma. Generally, it is associated to a pseudoaneurysm of the MAIVF (p-MAIVF) or aortic abscesses [1]. MAIVF fistulas could potentially lead to devastating complications and a high mortality rate; additionality, the defect is related to severe regurgitation due to aortic valve dehiscence [2]. Clinical features may vary depending on the underlying cause, the spectrum ranges from asymptomatic patients to cases presenting fever spikes and symptoms of congestive heart failure along with pulmonary edema [2, 3]. Most commonly, cases are identified incidentally during an echocardiographic evaluation, in which case a transesophageal echocardiogram (TEE) would optimize characterization of the condition. The selection of the approach for the surgical management, either percutaneous or open closure, depends on the clinical presentation, structural valve damage and/or presence of vegetations [1]. Herein we describe the complex case of a patient with a MAIVF fistula secondary to endocarditis by *Streptococcus Anginous* which provoked severe aortic valve insufficiency. The condition was not associated to previously observed p-MAIVF or aortic abscess. Surgical repair and closure were indicated.

## Case presentation

**Fig. 1:**
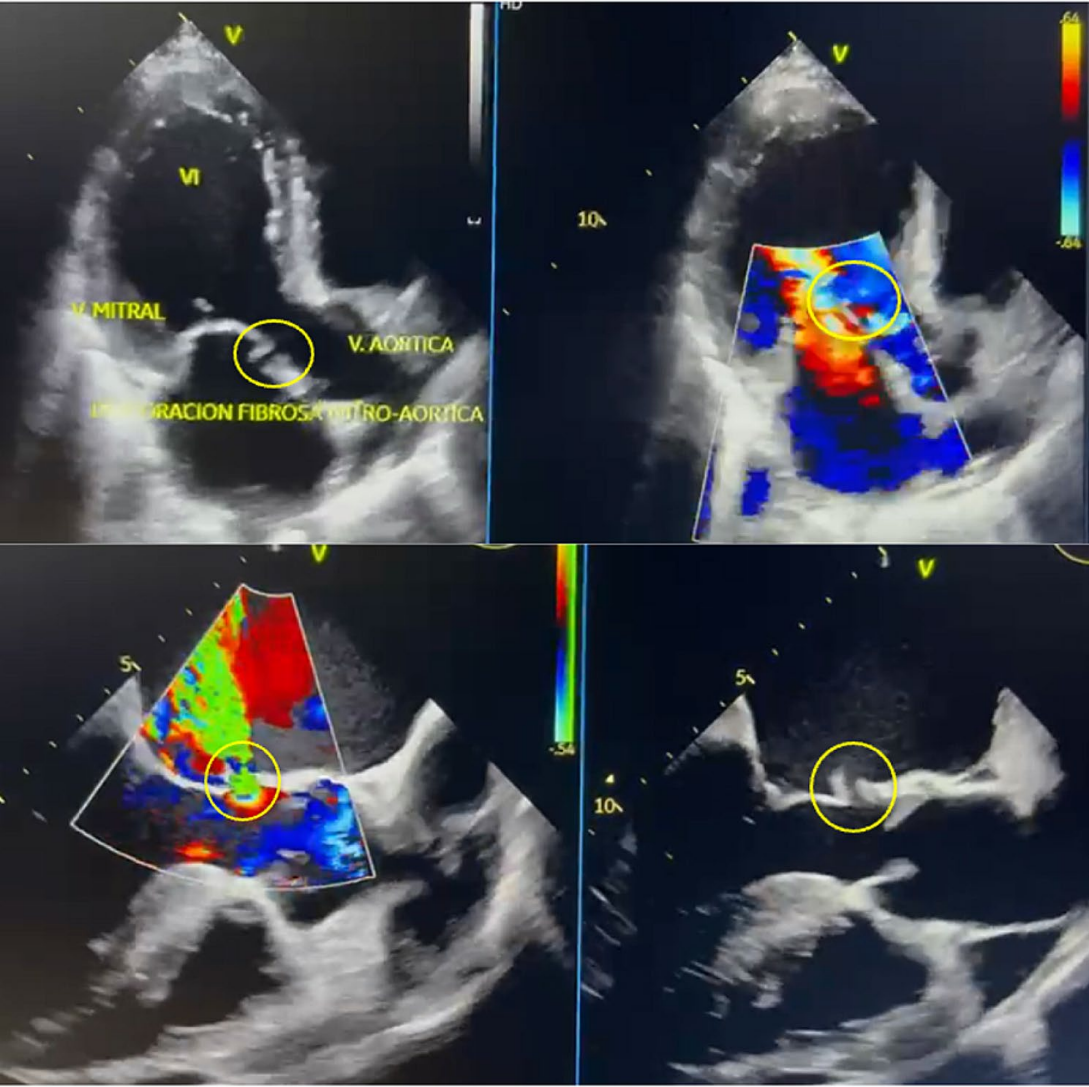
Preoperative transesophageal echocardiogram **(A)** Two-chamber apical view. Evidence of fistula in the mitro-aortic fibrous portion (yellow circle) with competitive flow. **(B)** Longitudinal or long-axis view. Evidence of fistula in the mitro-aortic fibrous portion (yellow circle) with competitive flow.

A 74-year-old male patient was admitted to a primary care center with complaints of malaise, asthenia, adynamia, hyporexia and lower limb edema over the past eight days. His past medical history is positive for arterial hypertension and being monorenal. Decompensated heart failure was suspected; thus, the patient was transferred to our high complexity medical center where he would be assessed by the internal medicine department. Upon arrival severe lower limb edema was observed; therefore, blood samples for laboratory tests were withdrawn, revealing an elevated NT-proBNP (7620 pg/ml), abnormal nitrogen levels (Creatinine 1.46 mg/dL and urea nitrogen 28.3 mg/dL). Additionally, a chest X-ray showed evidence of cardiomegaly and pulmonary edema. The case was assessed by the cardiology team, who concluded that the clinical presentation was compatible with congestive heart failure, characterized as class IV according to the New York Heart Association (NYHA) and as Stevenson C for the hemodynamic profile. Pharmacological management consisting of furosemide, spironolactone, dapagliflozin and sacubitril/valsartan, along with the conduction of a TEE (Fig. 1) were deemed necessary. The TEE exhibited 56% left ventricle ejection fraction (LVEF) and a complicated aortic valve endocarditis: Severe insufficiency due to the rupture of the left coronary leaflet, along with the presence of a mitro-aortic fistula with flow from the outflow tract to the left atrium), associated with bi-atrial dilation and moderate tricuspid insufficiency caused by a probable rupture of the anterior valve, which is why surgical management through cardiovascular surgery was recommended (Fig. 1). Coronary angiography was conducted to rule out secondary coronary artery disease, and no significant lesions were found in the epicardial coronary arteries.

Parallelly, blood cultures confirmed the presence of *Streptococcus Anginous* leading to the infectious disease team determination to suspend broad-spectrum empirical antibiotherapy and establish a new regime with ceftriaxone for 28 days. Considering the structural compromise observed in the TEE and etiologic confirmation of bacteriemia, a definite diagnosis of infective endocarditis was appropriate. Furthermore, contemplating the extent of the structural defect and hemodynamic consequences, surgical management including vegetation resection, valve replacement and closure of MAIVF fistula was indicated. Additionally, the structure of the tricuspid valve was assessed, as it could be managed by repairing its leaflet through tricuspid valve plasty or, alternatively, tricuspid valve replacement.

**Fig. 2:**
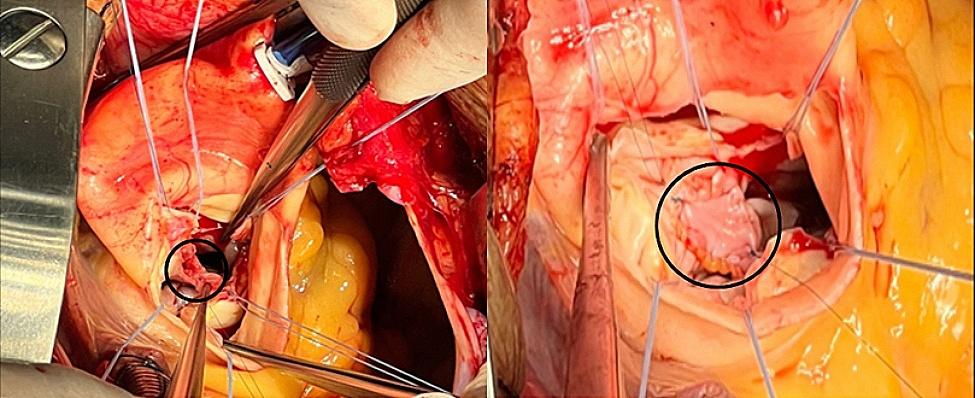
Intraoperative view of the MAIVF fistula **(A)** Fistula located in the mitro-aortic fibrous portion (Black circle) resected through a left ventricular approach. **(B)** Mitro-aortic fistula sealed with an autologous pericardial patch (Black circle).

**Fig. 3:**
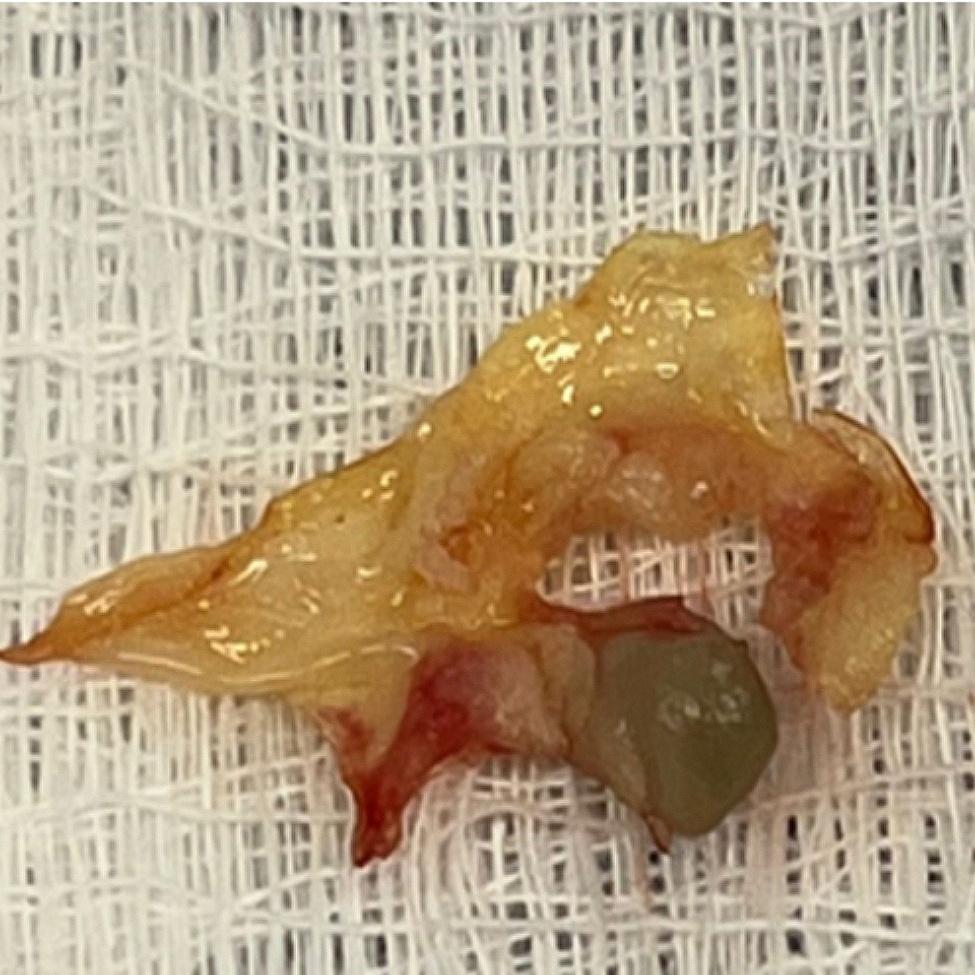
Surgical specimen

Surgical intervention was conducted with extracorporeal circulation support, featuring pump and clamp times of 120 min and 89 min, respectively. Initially, the aortic valve was excised and extracted (Fig. 2), followed by the closure of the mitro-aortic fistula using an autologous pericardial patch (Fig. 3). Subsequently, an aortic valve replacement was performed using a 25 mm bioprosthesis, The anatomical structure of the tricuspid valve was assessed, revealing no involvement of the chordae tendineae of the anterior leaflet. An in vivo mechanical test was also conducted, which did not show retrograde flow in the tricuspid valve. Therefore, neither valve repair nor replacement was deemed necessary. As a result, the patient was decannulated, and layered closure was performed. The patient was transferred to intensive care unit (ICU) under orotracheal intubation and vasoactive support was provided with low doses of noradrenaline and vasopressin. The infectious disease team recommended a 28-day continuation of antibiotherapy with Ceftriaxone.

Surgical intervention was conducted with extracorporeal circulation support, featuring pump and clamp times of 120 min and 89 min, respectively. Initially, the aortic valve was excised and extracted (Fig. 2), followed by the closure of the mitro-aortic fistula using an autologous pericardial patch (Fig. 3). Subsequently, an aortic valve replacement was performed using a 25 mm bioprosthesis, The anatomical structure of the tricuspid valve was assessed, revealing no involvement of the chordae tendineae of the anterior leaflet. An in vivo mechanical test was also conducted, which did not show retrograde flow in the tricuspid valve. Therefore, neither valve repair nor replacement was deemed necessary. As a result, the patient was decannulated, and layered closure was performed. The patient was transferred to intensive care unit (ICU) under orotracheal intubation and vasoactive support was provided with low doses of noradrenaline and vasopressin. The infectious disease team recommended a 28-day continuation of antibiotherapy with Ceftriaxone.

Surgical intervention was conducted with extracorporeal circulation support, featuring pump and clamp times of 120 min and 89 min, respectively. Initially, the aortic valve was excised and extracted (Fig. 2), followed by the closure of the mitro-aortic fistula using an autologous pericardial patch (Fig. 3). Subsequently, an aortic valve replacement was performed using a 25 mm bioprosthesis, The anatomical structure of the tricuspid valve was assessed, revealing no involvement of the chordae tendineae of the anterior leaflet. An in vivo mechanical test was also conducted, which did not show retrograde flow in the tricuspid valve. Therefore, neither valve repair nor replacement was deemed necessary. As a result, the patient was decannulated, and layered closure was performed. The patient was transferred to intensive care unit (ICU) under orotracheal intubation and vasoactive support was provided with low doses of noradrenaline and vasopressin. The infectious disease team recommended a 28-day continuation of antibiotherapy with Ceftriaxone.

Resected aortic valve with a vegetation secondary to bacterial endocarditis.

**Fig. 4:**
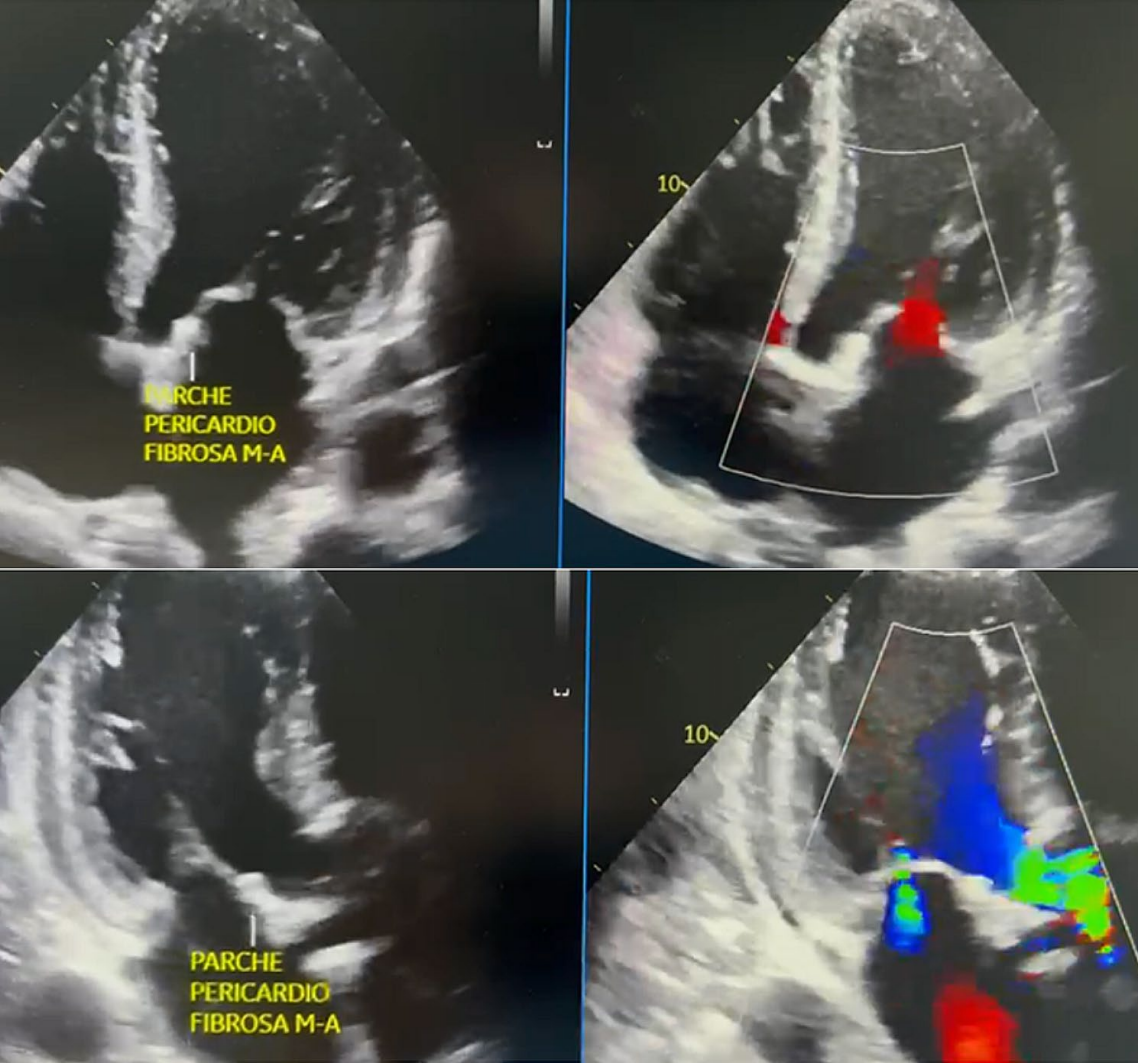
Postoperative transesophageal echocardiogram **(A)** Four-chamber apical view. Evidence of pericardial patch in the mitro-aortic fibrous portion occluding the fistulous tract, with no flow. **(B)** Two-chamber apical view. Evidence of pericardial patch in the mitro-aortic fibrous portion occluding the fistulous tract, with no flow.

Two days postoperatively, a follow-up TTE was performed, revealing an ejection fraction of 26%, a normally functioning #25 aortic bioprosthesis, mild secondary mitral insufficiency, and mild tricuspid valve regurgitation. Extubation proceeded without complications, and vasopressor support was discontinued. On the seventh postoperative day, the patient was transferred to the hospital, presenting hypotensive blood pressure readings. Consequently, an urgent TEE was recommended, confirming a LVEF of 26%, functional #25 aortic bioprosthesis with no evidence of mitro-aortic fistula (Fig. 4). Additionally, a severe circumferential pericardial effusion of approximately 520 ml with signs of cardiac tamponade was noted. Urgent pericardiocentesis was performed, draining 150 cc of hematic fluid, and a catheter was left in place for continued drainage.

Consequently, the patient was readmitted to the intensive care unit, and a gradual adjustment of hemodynamic support was initiated, leading to a progressive improvement. Ultimately, after 30 days postoperatively and upon completing the aforementioned antibiotic regimen, the decision was made to discharge the patient with medical orders. At the 30-day follow-up appointment with our specialty, a review of the cardiac Holter report revealed no evidence of arrhythmias. The echocardiogram showed the absence of a mitro-aortic fistula, a normally functioning aortic bioprosthesis with an ejection fraction of 35%, cardiovascular asymptomatic status, and good tolerance to physical activity. Thus, the patient was discharged from cardiovascular surgery care. However, continued follow-up under cardiology supervision persisted until 8 months post-procedure, during which a transthoracic echocardiogram reported an ejection fraction of 50%, a normally functioning biological prosthesis in the aortic valve position. During the clinical evaluation the patient indicated NYHA class I-II symptoms.

### Systematic literature review

**Fig. 5:**
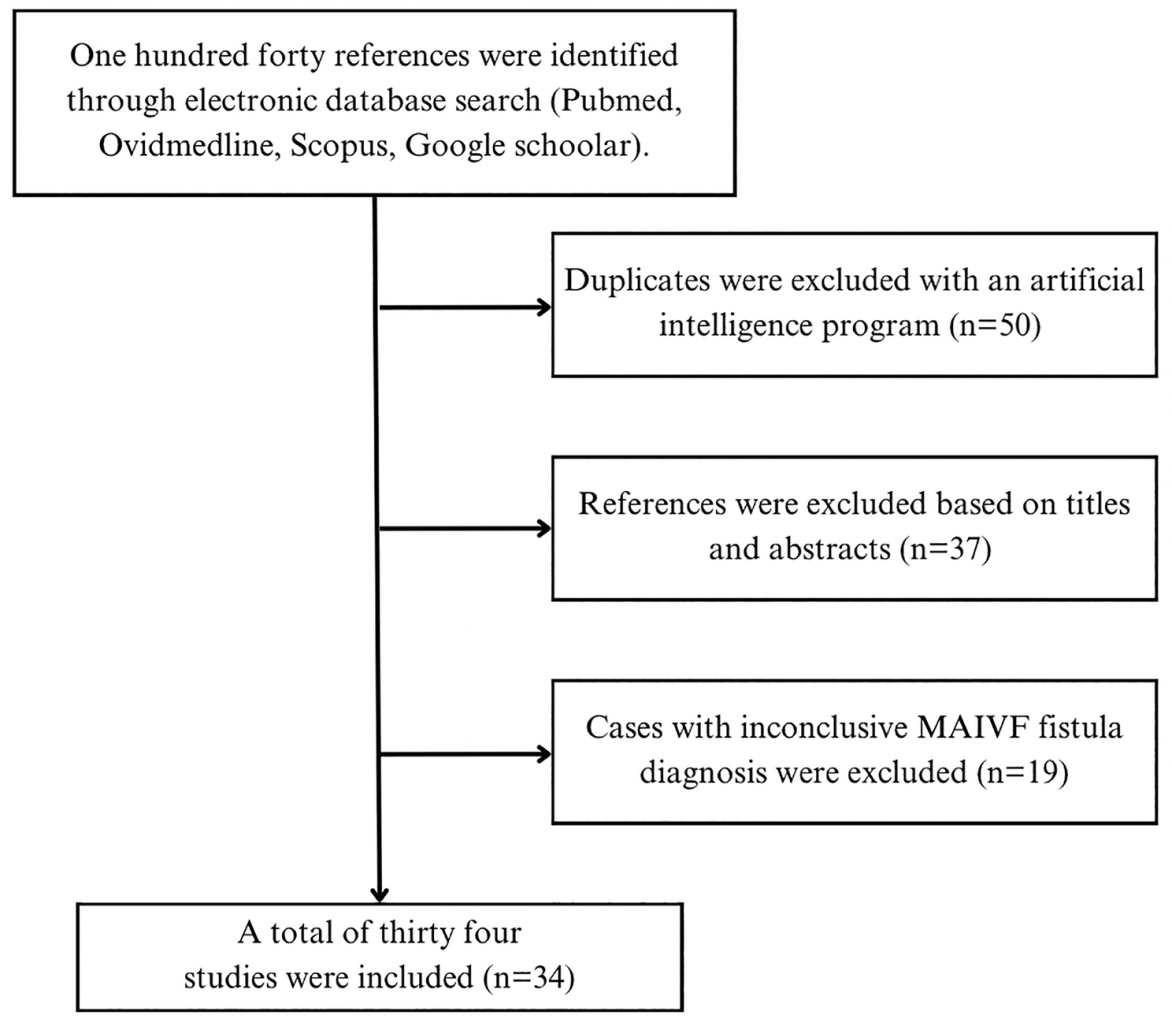
Flowchart illustrating study selection process

A systematic literature review was carried out in order to identify the available resources pertaining to the clinical case. 1 were used by applying a search strategy consisting of keywords and pre-established criteria of inclusion. All authors agreed on the application of a multi-string search strategy with the terms: “(mitral-aortic fibrosa) AND (fistula)”, “(intervalvular fibrosa) AND (fistula)”, “(mitral-aortic junction) AND (fistula), ((aortomitral) OR (aorto-Mitral) or (Mitro-Aortic) AND fistula)). The search was restricted to articles written in English and Spanish, without additional filters for the publication date. Articles herby included were published before January 16th, 2023. Methodological quality of the obtained publications was assessed by the Grading of Recommendations, Assessment, Development and Evaluation (GRADE). Figure 5 exhibits a flowchart of the study selection process.

Even though transversal studies were identified; information was insufficient for the analysis of MAIVF fistula specific presentation and outcomes. Additionally, no controlled randomized trials or controlled cohort studies were retrieved. Thus, thirty-four case reports about MAIVF fistulas were scrutinized and the pertinent demographical and clinical features were summarized on Table 1 (available in the document “Additional file 1”).

Case-reports on the matter were retrieved from a diverse spectrum of countries. Notably, reports from Latin American countries were not identified, which would imply that the clinical case hereby presented will be the first report of its kind in our region. The mean age at the time of diagnosis was 50.06 years old, with a range between 20 and 81 years old. The ratio male to female was 29:5. Importantly, eighteen patients had a past medical history of cardiovascular surgery and previous infective endocarditis caused by microorganisms such as *Corynebacterium, Group C beta hemolytic Streptococcus and Neisseria gonorrhea* [4–6].

MAIVF fistula has been described as a rare and critical complication of infective endocarditis (IE); however, our research points out that such a structural defect could also be attributed to a surgical complication. 67.6% (*n* = 23) of the cases were confirmed to be associated to IE. The microbiological agents were distributed as follows: *Staphylococcus aureus* was detected in eight cases (47.1%) [1, 7–11]. 29.4%(*n* = 5) of the cases were caused by microorganisms from the *Streptococcus* genus [12–16]. One exceptional case was caused by *enterococcus* [17]. Interestingly, three cases were taken as the complication of an IE that occurred months prior [18–20]. MAIVF fistulas associated to prior cardiac interventions were confirmed in five clinical cases; time interval from the surgical procedures to the new event were significantly different between cases.

Concerning the therapeutic approach exhibited in the review, 90% of authors considered surgical intervention as the most appropriate management for each patient. 42.9% of the interventions involved patch repair; including bovine, porcine and unspecified origin patches were used [4, 5, 7, 8, 11, 21, 22]. Repair with an autologous patch was described in only one reference [5]. More than 57% of surgical cases required mitral and/or aortic valve replacement.

Most commonly, fistulous tracts in the MAIVF are observed in association to other morphological and functional changes. As exhibited by this case, fistulous tracts may be present in the absence of other defects. Interestingly, MAIVF fistulas and pseudoaneurysms may lead to compression of structures. For instance, 3D TTE and angiography revealed a fistulous communication in the MAIVF, a pseudoaneurysm in the same region associated to systolic compression and narrowing of the left main trunk [18].

Postoperative (POP) outcomes are favorable in most cases, 73.9% of the surgical interventions were successful throughout follow-up. Negative results included dehiscence of the MAIVF patch and the subsequent recurrence of the fistula in one case, and early mortality (first 24 h after the procedure) due to multiorgan failure, malignant ventricular arrythmia. Notably, the clinical case in which antibiotherapy without surgical intervention was preferred resulted in mortality [23].

## Discussion

The MAIVF is located between the anterior leaflet of the mitral valve and the non-coronary and left coronary cusps. A mitral aortic fistula is described as the presence of an anomalous pathway within this region. Most frequently, the occurrence of this structural defect is associated to a preexisting or concomitant pseudoaneurysm, which is referred as p-MAIVF. Up to 20% of the patients with p-MAIVF also develop fistulas at this level [24], phenomenon which was further reinforced by the case reports we reviewed [1, 7]. However, in the case we described the TEE nor direct intraoperative observation led to the identification of a pseudoaneurysm in this region. Meaning that this report represents a unique clinical presentation of a rare condition, an isolated MAIVF fistula [1, 3, 7]. What is more, structural complications secondary to the presence of the p-MAIVF have been described in the literature; they include coronary artery occlusion, cardiac tamponade, and intra-aneurysmal thrombosis with the potential to cause systemic embolism and stroke. None of these were present in our clinical case, further reinforcing its peculiarity.

Having established that most of MAIVF fistulas are the result of a ruptured pseudoaneurysm; it is worth detailing that IE and previous aortic valve surgery are the most common etiologies of pseudoaneurysms in the fibrosa. The underlying mechanism for this condition articulates two concepts: 1). The MAIVF is an avascular tissue, which conditions higher propensity to infections. 2). The cardiac cycle generates shear forces that provoke remodeling of the fibrosa. Both events take place when there is an aortic valve infection or by the surgical replacement of the valve. However, congenital defects of the MAIVF and blunt thoracic trauma are less frequent conditions that might lead to the formation of p-MAIVF [21, 25]. Particularly, in the clinical case herein presented the patient was diagnosed with bacterial endocarditis due to *Streptococcus Anginous*, which was presumed as the direct cause of the fistula even though there was no previous history of valve surgery. The latter differentiates this case from other case reports; where patients presented infective endocarditis by Staphylococcus Aureus summed to a past medical history of valvular surgery [1, 7].

An accurate diagnosis of life-threatening structural defects of the heart will likely increase the success of a surgical intervention and patient survival. Therefore, cardiac diagnostic imaging used on patients with suspected valvular anomalies should yield the highest sensitivity possible. TEE has shown of 90%, which corresponds to a higher diagnostic power compared to transthoracic echocardiography (TTE). In our review, the diagnostic tool most frequently used was the TEE. It should be noticed that recent technological advances developed new strategies such as color flow doppler and 3D rendition to maximize the sensitivity of this imaging modality. Three of the clinical cases were diagnosed by TEE with color flow doppler [5, 6, 12]. Despite being less frequently observed on the reports, TTE and TTE enhanced with 3D technology and color doppler was also reported on the literature. In specific scenarios ventriculography and aortography were utilized to make a preoperative diagnosis, yet, in one particular case an intraoperative view was necessary to confirm the presence of a MAIVF fistula. In addition to the TEE, diagnostic imaging techniques such as computed tomography (CT) and cardiac magnetic resonance (CMR) could be used to characterize the lesion, identify potential complications, and direct the therapeutic approach [26, 27].

The definite treatment of a fistulous tract in the MAIVF involves the prompt closure of the defect, which can be achieved through either a percutaneous or an open surgical approach. Failure to correct this structural abnormality in a timely manner can lead to a range of adverse clinical outcomes. These may include hemodynamic instability, septic shock, cardiac tamponade and, in severe cases, may even result in a fatal outcome. Such is the case of the patient reported by Fazlinezah et al., the aortography confirmed the presence of a large fistula between the aortic root and left ventricular outflow tract, and severe aortic regurgitation [28]. Unfortunately, the patient died due to rapid decompensation and insufficient time for OR transport. Additionally, to the selection of the surgical approach, the team must decide which type of patch will be used for closure. In our case, similarly to Spampinato 2012, an open surgical repair was achieved by an autologous pericardial patch. Kahraman 2023 et al. were also inclined for an open surgical approach, but in contrast to our case, a porcine pericardial patch was used. Another occlusive method involves an Amplatzer Vascular Plug II, its use is described in a patient with history of two cardiac interventions without associated valvopathy [1, 3, 7].

In general terms, postoperative follow-up is focused on the evaluation of cardiac symptoms and tolerance to physical activity, interpretation of a Holter report in order to exclude arrythmias and the observation of structural and hemodynamic parameters on the echocardiogram. The case reports we reviewed confirmed at follow-up improvement or resolution of symptoms, as well as the absence of residual flow tracts and valve dysfunction [3, 7]. Similarly, our clinical case had a favorable clinical evolution. The patient was asymptomatic at the time, the Holter did not exhibit significant arrythmias and the TEE reported 35% LVEF without MAIVF fistula. It is worth mentioning that not all outcomes are as fortunate; during the postoperative monitoring of a percutaneous closure, the TEE only showed a mild decrease of pseudoaneurysm flow [1].

## Conclusion

This systematic review provides valuable information and insights into the demographics and clinical features of MAIVF fistulas. Additionally, this manuscript reinforces the significance of accurate diagnostic imaging modalities and their impact in the timely medical attention. From a surgical standpoint, this review highlights the technical complexity embedded in the reconstruction of cardiac tissue, especially, when there are multiple fibrous structures involved. Despite the identification of a substantial number of case-reports, further research should include enhanced epidemiological methodologies and larger populations. This will allow a more comprehensive understanding of MAIVF fistula and its surgical implications.

This case is unique because the fistula occurred in the absence of a p-MAIVF and an aortic abscess; furthermore, the patient was endangered by the subsequent severe aortic valve insufficiency. A prompt closure was attained by the placement of an autologous pericardial patch during an open surgical approach. Further epidemiological and clinical studies with robust methodologies are required to fully elucidate this entity. Nevertheless, this clinical case contributes with the existing body of literature and reinforces the importance of timely diagnosis and appropriate treatment selection in complex and potentially mortal conditions such as this one.

### Electronic supplementary material

Below is the link to the electronic supplementary material.

Supplementary Material 1

## Data Availability

Availability of the data used and analyzed during the writing of the case report is under the responsibility of the corresponding author, and its distribution is authorized upon reasonable request.

## Abbreviations

CBP: Coronary Bypass Time
CMR: Cardiac Magnetic Resonance
CT: Computerized Tomography
ICU: Intensive Care Unit
IE: Infective Endocarditis
LVEF: Left Ventricular Ejection Fraction
MAIVF: Mitro-Aortic Intervalvular Fibrosa
NYHA: New York Heart Association
OR: Operating Room
p-MAIVF: Pseudo Aneurysm of the Mitro-Aortic Intervalvular Fibrosa
POP: Postoperative
Pro-BPN: Pro–B-Type Natriuretic Peptide
TEE: Transesophageal Echocardiogram
TTE: Transthoracic Echocardiogram
