# Supplementary material for: Fistula of the mitral-aortic intervalvular fibrosa in a patient with bacterial endocarditis: a case report and systematic literature review
Source: J Cardiothorac Surg. 2024 May 28;19:300. doi: 10.1186/s13019-024-02736-5 (PMC11131286; doi:10.1186/s13019-024-02736-5)
Supplement: Supplementary file 1 — Supplementary Material 1 [file 13019_2024_2736_MOESM1_ESM.docx]

**Table 1. Demographical and clinical information pertaining to the clinical cases of MAIVF fistula.**

| **Clinical Case (Author, year of publication)** | **Publication´s Country of Origin** | **Patient´s demographic Age (years), sex** | **Previous cardiac Condition or Intervention** | **Diagnostic Method** | **Associated cardiac findings** | **Etiology** | **Therapeutic approach** | **POP-follow up** |
| --- | --- | --- | --- | --- | --- | --- | --- | --- |
| Cakal et al, 2021 | Turkey | 76, female | Yes: redo aortic valve replacement. | TEE | P-MAIVF | Methicillin-sensitive Staphylococcus aureus confirmed IE. | Percutaneous closure, Amplatzer vascular plug II deployment. | Asymptomatic at the six-month follow-up. |
| Silbiger, 2013 | USA | 54, male | Yes, infective endocarditis of the mitral valve due to Group C BH streptococcus. Treated with antibiotherapy | TEE and color flow doppler. | P-MAIVF and fistulous communication with the LA. | Intraoperative findings suggestive of prior endocarditis. | Posterior leaflet was surgically detached to allow primary closure of the ventricular opening, and then reattached to the annulus using a patch of autologous glutaraldehyde-fixed pericardium, and the whole repair stabilized with an annuloplasty ring. | POP TEE confirmed no residual mitral regurgitation and obliteration of the pseudoaneurysm. |
| Kuroda, 2020 | Japan | 20, male | No | TEE and color flow doppler; confirmed by contrast enhanced CT. | p-MAIVF, pericardial effusion | Methicillin-sensitive Staphylococcus aureus, confirmed IE. | Complete abscess debridement with vegetation removal and closure by Dacron patch. Surgical drainage of purulent hemorrhagic pericardial fluid. Antibiotherapy | NA |
| Kahraman et al, 2022 | Turkey | 63, male | Metallic AV and MV replacement. | TEE. | P-MAIVF | Methicillin-resistant Staphylococcus aureus (MRSA); confirmed IE. | Antibiotherapy.  Excision of infected AV and MV, MAIVF segment and P-MAIVF.  Double valve replacement and porcine pericardial patch. | No valvular or ventricular dysfunction in the 6^th^ month POP TEE. |
| Sheppard, 1991 | United States | 28, male | Aortic valve replacement for acute aortic insuffiency due to gonococcal infective endocarditis | TEE with color flow doppler | No | Enterococcus sensitive to ampicillin. Confirmed IE. | Repeated aortic valve replacement. | POP TEE: smaller abscess, non-pulsatile, no communication between the abscess cavity and LVOT. |
| Goodwin et al, 2019 | United States | 27, male | No | TTE | No | Methicillin-resistant Staphylococcus aureus (MRSA); confirmed IE | Bioprosthetic aortic valve replacement, porcine aortic root reconstruction, and patch repair of the left atrium and MAIVF. | 3 weeks following surgery developed ARDS, repeated TTe showed dehiscence of the MAIVF patch and recurrence of MAIVF fistula. |
| Pereira et al, 2014 | Portugal | 72, male | No | TEE | P-MAIVF | penicillin-sensible Streptococcus pneumonia.  confirmed IE | Surgery not specified. | Died 24h after surgery due to multiple organ dysfunction. |
| Agrawal et al, 2016 | Unites States | 57, male | No | TEE | No | Staphylococcus aureus; confirmed endocarditis. | Deceased before surgery | NA |
| Abdallah et al, 2017 | Canada | 62, male | 1). mechanical mitral and aortic valve replacements; 2). 2 years later: endocarditis- MAIVF repair with bovine pericardial patch. | TEE | P-MAIVF | No signs of active endocarditis; hence, considered a complication following MAIVF repair. | Removal of previous bovine patch, MAIVF reconstruction with new bovine patch. | Preserved prostheses function with no residual fistula through 16-month follow-up. |
| Hasebe et al, 2016 | Japan | 72, male | No | Left ventriculography and intraoperative observation | P-MAIVF | IE*. Streptococcus sanguini* | Antibitherapy, P-MAIVF repair, aortic valve replacement with bioprosthetic, fistula repair with patch. | Uneventful postoperative course. |
| Biswajit et al, 2015 | India | 40, male | No | TTE | P-MAIVF | Not stablished | Not stablished | Not stablished |
| Fazlinezhad et al, 2012 | Iran | 62, male | no | TEE and aortography | P-MAIVF | Coagulase positive Staphylococci. confirmed IE. | Deceased before transfer to the OR. | NA |
| Bagh et al,l 2021 | United States | 49, male | Yes, Bentall procedure 3 years earlier. | TEE and color doppler | P-MAIVF, fistulous communication between LV and LA. | Group B S*treptococcus.* confirmed IE. | Antibiotherapy, diuretics and unspecified surgical management. | Not disclosed |
| Giannocaro et al, 1991 | Canada | 51, male | no | TEE | P-MAIVF | [coagulase-positive staphylococcus. confirmed endocarditis.](https://doi.org/10.1016/S0894-7317(14)80450-1) | Antibiotherapy | After 3 weeks at home required readmission for worsening HF, died despite aggressive medical therapy. |
| Kruithoff et al, 1995 | United States | 34, female | No | TEE | P-MAIVF | Complication of previous MAIVF repair | Intervalvular fibrosa obliteration by attaching aortic prosthesis to the mitral valve sewing ring. | POP TEE: normal prosthetic valve function. Primary repair followed by redo of the mitral valve replacement. 8 months POP – asymptomatic. |
| Yong et al, 2015 | Australia | 51, male | Yes: history of *Corynebacterium* endocarditis that had affected his native bicuspid aortic valve (replaced, 5 months earlier, with a bio prothesis. | TEE with 3D rendition | Mycotic aneurysm | *IE. Hemophilus parainfluenza*. | Concomitant aortic and mitral valve replacement, reconstruction of LVOT and intervalvular fibrosa by bovine pericardial patches. | POP TTE: satisfactory valve function. |
| Chandra el al, 2013 | India | 20, male | No. | 3D TTE with color doppler | No | Methicillin-resistant Staphylococcus aureus | Repair of fistula with aortic valve replacement and mitral valve repair. | Successful immediate POP. No further follow-up. |
| Kunavarapu et al, 2008 | United States | 59, male | No. | TEE | Aneurysm | Methicillin-sensitive Staphylococcus aureus | Pericardial patch to close the fistula and repair the aneurysm | Not disclosed |
| Grande, 2017 | Italy | 66, male | Yes, mitral-aortic endocarditis. | 3D TTE and angiography. | P-MAIVF, systolic compression and narrowing of the left main trunk. | Complication of previous mitral and aortic valve replacement. | Infected tissues and previous implants were removed and replaced. | Uneventful postoperative course. TTE showed normal bio prostheses function 1 year later. |
| Agirbasli, 1999 | USA | 52, male | Yes, aortic valve replacement with a mechanical prosthetic valve secondary to IE. | Multiplane TEE with color doppler. | P-MAIVF; pulsatile 6 x 7 cm anterior chest wall mass | Long-term complication of aortic valve endocarditis. | Excision of pseudoaneurysm with reconstruction of the intervalvular fibrous body. | POP TEE: restoration of the normal mitral-aortic continuity. |
| Stechert, 2007 | USA | 65, male | Yes, aortic valve replacement for aortic insufficiency due to IE. | TTE, TEE and color flow doppler. | Bulging interatrial septum with patent foramen ovale, left to right shunt. | Aortic prosthetic valve endocarditis | Removal of the infected valve and replacement by a bileaflet mechanical valve. Obliteration of fistula tracks. Closure of foramen ovale by patch repair. | Repair was confirmed by TEE. |
| Apostolidou, 2017 | USA | 67, male | Aortic valve replacement with a bovine pericardial valve and patch repair of the aortic abscess. | TEE. | P-MAIVF and fistula to the left atrium. | Late complication of IE. | Repeat aortic valve replacement with a tissue valve and repair of the pseudoaneurysm. | Unremarkable POP course. 4 month follow up: persistent P-MAIVF. |
| Nakata, 2023 | Japan | 65, male | Asymptomatic moderate aortic valve regurgitation. | TEE and TTE | P-MAIVF and backflow into the left atrium. | Aortic valve regurgitation and IE (not confirmed). | Aortic valve replacement with bioprosthetic pericardial tissue, mitral valve annuloplasty and P-MAIVF repair with a bovine pericardial patch. | Uneventful POP. |
| Mohseni-Badalabadi (Hosseinsabet) | Iran | 55, male | Aortic and mitral valve replacement with mechanical bileaflet valves.­ | TTE and TEE. | P-MAIVF connected to the aorta on one side and to the left atrium on the other side. | Surgical trauma from previous intervention. | The patient refused surgical repair. | NA |
| Chiocchy, 2021 | Italy | 81, female | Aortic valve replacement using biological prosthesis. | TTE, Coronary Computed Tomography Angiography. | P-MAIVF, connected with the right ventricular outflow tract through a fistula. Compression of right coronary artery by mycotic pseudoaneurysm. | IE. | Antibiotherapy. Team advised against surgery. | Follow up after 6 months with cardiac CT confirmed the presence of pseudoaneurysm, fistula and compression of RCA. |
| Çimen, 2015 | Turkey | 44, male | NA | 2D-TTE and TEE. | MAIVF pseudoaneurysm and fistulization toward the left atrium. | IE by Streptococcus. | MAIVF pseudoaneurysm restored using bovine pericardium patch, aortic valve replacement.  Antibiotherapy. | NA |
| Di Cori, 2012 | Italy | 74, male | NA | TTE and color doppler. | Ruptured-flailing mitral-aortic intervalvular fibrosa, fistula to LVOT and LA | IE by Listeria monocytogenes. | Flailing tissue was resected, followed by direct continuous suture reinforced with pledged mattress sutures. | Recovery without periprocedural complications. |
| Varga, 2021 | Romania | 43, female | Yes, Bicuspid aortic valve and severe valvulae aortic stenosis. | TTE, TEE and color flow doppler, contrast enhanced MDCT | P-MAIVF, communication between LVOT and P-MAIVF | IE, unidentified microorganism. | Surgical intervention: Morrow classical septal myectomy; suture the ostium of the P-MAIVF; replacement of affected valve with mechanical prosthesis. | Uneventful POP curse for the first week. Sudden death on day 10 (before discharge), attributed to a malignant ventricular arrythmia. |
| Velibey, 2013 | Turkey | 80, male | Yes, bioprosthetic aortic valve replacement. | 2D TTE, 2D and 3D TEE | P-MAVIF and direct fistulous communication between P-MAIVF and LVOT. | IE by Enterococcus faecium | Urgent aortic surgery. | Developed ischemic stroke and multiple organ failure, died in the POP. |
| Xie, 2023 | China | 31, male | Yes, PTCA | TTE, contrast-enhanced CT and 3D-TEE | P-MAIVF and orifice of the pseudoaneurysm located between the left coronary valve, non-coronary valve and anterior mitral valve. | Injury of the cardiac catheter while crossing the aortic annulus. | Surgical repair of the aneurysm. | NA |
| Bansal, 1990 | USA | 45, male | Yes, AV replacement | 2D TTE and color flow doppler, TEE | Rupture of the MAIVF and direct shunting of blood from the LVOT to LA during systole. | IE | Antibiotherapy. Removal of AV, closure of fistulous communication with multiple pledgeted sutures. AV replacement. | Uneventful POP recovery. |
| Lacalzada-Almeida, 2020 | Spain | 68, male | No | TTE, 3D TEE | P-MAIVF, fistula connecting with the LA causing severe mitral regurgitation. | NA | MV and AV replacement. | Died few hours after surgery due to low cardiac output. |
| Saadia, 2023 | Pakistan | 49, male | No | TTE and intraoperative TEE | Ruptured MAIVF access and fistula between the aorta and left atrium. | IE | Dual valve replacement, aortic repair. | POP first-degree AV block. Eventually discharged after 18 days with complete recovery. |
| Tak, 2001 | USA | 56, female | No | TTE and TEE | P-MAIVF. Abscess in the area of the left and non-coronary sinuses, fistula connecting LVOT with the MAIVF. | IE by group D Streptococcus | Debridement of necrotic tissue, infiltration with antibiotics. Pericardial patch to repair fistula in the MAIVF. Excision and replacement of prosthetic valve. | Uneventful POP. |

IE: Infective endocarditis; TEE: transesophageal echocardiogram; TTE: transthoracic echocardiography; MA: Mitral Valve; AV: aortic valve; LA: Left atrium; P-MAIVF: pseudoaneurysm; POP: postoperative; MDCT: Multidetector computed tomography; LVG: Left ventriculography; PCTA: percutaneus transluminal coronary angioplasty

NA: not applicable.
